# Digital odontometric analysis for sex estimation in Egyptian adolescents

**DOI:** 10.1186/s12903-026-09486-x

**Published:** 2026-08-08

**Authors:** Shimaa M. Hadwa, Safa B. Alawy, Ibrahim A. Kabbash, Shaimaa S. EL-Desouky

**Affiliations:** 1 Pediatric Dentistry, Oral Health, and Preventive Dentistry Department, Faculty of Dentistry, Tanta University, Tanta, 31527 Egypt; 2 Orthodontics Department, Faculty of Dentistry, Tanta University, Tanta, Egypt; 3 Public Health & Community Medicine Department, Faculty of Medicine, Tanta University, Tanta, Egypt

**Keywords:** Forensic odontology, Sex, Arch width, Odontometric analysis, Sexual dimorphism

## Abstract

**Objectives:**

Reliable sex estimation using dental metrics has important implications for clinical assessment and forensic identification, particularly in adolescents where skeletal indicators may be limited. This study aimed to validate the accuracy and clinical applicability of a digital odontometric model for sex estimation in an Egyptian adolescent population.

**Methods:**

A total of 150 dental casts from individuals aged 12–19 years were digitized using a high-resolution three-dimensional scanner. Mesiodistal and buccolingual dimensions of the permanent first molars, mesiodistal widths of the maxillary canines, and intercanine, interpremolar, and intermolar arch widths were obtained using Facad Ortho tracing software. Statistical comparisons between sexes were performed, and discriminant function analysis was applied to construct and validate a predictive model for sex estimation.

**Results:**

All measured parameters demonstrated significantly higher mean values in males compared with females (*p* < 0.05). The buccolingual and mesiodistal dimensions of the maxillary first molar exhibited the greatest sexual dimorphism (26.95% and 25.98%, respectively). The developed discriminant function model achieved a prediction accuracy of 97.8% for males and 100% for females.

**Conclusions:**

Digital odontometric analysis provides a promising approach for assessing sexual dimorphism in adolescents, pending external validation. This promising model may serve as a reliable adjunct for clinical evaluation and forensic identification within population-specific contexts pending further multicenter validation.

**Clinical relevance:**

Digital measurement of tooth and arch dimensions enables objective, standardized assessment of sex-related differences, supporting its integration into orthodontic diagnostics, treatment planning, and forensic casework where conventional skeletal indicators may be unavailable or inconclusive.

**Supplementary Information:**

The online version contains supplementary material available at 10.1186/s12903-026-09486-x.

## Introduction

Reliable identification of human remains is a fundamental requirement in both routine forensic casework and mass casualty incidents, including transportation accidents and natural disasters. Beyond its legal and humanitarian importance, human identification also represents a core area of investigation within physical anthropology and forensic sciences [1].

Forensic identification strategies are broadly categorized into comparative and reconstructive frameworks. Comparative approaches rely on matching postmortem findings with available antemortem records using primary identifiers such as genetic profiles, dental characteristics, and friction ridge patterns, often enabling a high level of certainty in individualization [2]. In the absence of antemortem documentation, reconstructive methods are employed to establish a biological profile, typically encompassing age, ancestry, stature, and sex. Among these components, sex assessment is generally regarded as a critical initial step that narrows the range of possible matches and guides subsequent analytical procedures. A variety of techniques have been described for this purpose, including lip print analysis, dental metric evaluation, skeletal measurements, and molecular-based testing [3–6].

Although genetic analysis provides a high degree of accuracy and comprehensive biological information, its application may be constrained by financial cost, infrastructure requirements, and the need for specialized technical expertise, particularly in resource-limited settings [7]. These limitations have encouraged continued interest in alternative approaches based on skeletal and dental characteristics that are more accessible and less resource-intensive.

Recent advancements in digital workflows, particularly three-dimensional imaging and cone-beam computed tomography (CBCT), have revolutionized the field of forensic odontology [8]. These technologies not only enhance the accuracy of measurements but also facilitate the application of machine learning techniques for sex estimation [9]. The integration of digital methods allows for a more nuanced analysis of dental morphology and sexual dimorphism, providing a more comprehensive understanding of population-specific variations [10].

Dental tissues are among the most resilient structures in the human body, demonstrating strong resistance to postmortem degradation and environmental insult. Once fully formed, teeth do not undergo physiological remodeling, making them valuable repositories of biological information over extended periods [11]. Several dental-based strategies for sex estimation have been reported, ranging from chromosomal analysis of dental pulp to optical and physical assessments of enamel and dentin properties [12].

Metric analysis of tooth dimensions, or odontometry, has gained particular attention due to its simplicity, reproducibility, and minimal equipment requirements. Consistent patterns of sexual dimorphism have been observed in tooth size, with male dentitions generally exhibiting larger dimensions than those of females, although the magnitude of this difference varies according to tooth type and population group [11–13].

A wide range of odontometric variables has been explored in previous studies, including measurements of canines and molars in both arches, as well as transverse arch widths such as intercanine, interpremolar, and intermolar distances [4, 14–16]. The first permanent molars are of particular interest because of their early eruption and relatively low frequency of impaction, while canines, especially in the maxillary arch, have frequently demonstrated pronounced dimorphic characteristics [4]. Importantly, dental morphology and size are influenced by genetic background and environmental factors, leading to population-specific variation in the reliability and applicability of proposed sex estimation models [17].

Accordingly, the present study aimed to evaluate sexual dimorphism in a sample of Egyptian adolescents using odontometric measurements of permanent teeth and dental arch dimensions. A secondary objective was to develop a population-specific predictive equation for sex estimation based on the most discriminative parameters. It was hypothesized that measurable sex-related differences exist within the studied dental variables and that these differences can be utilized to construct a reliable predictive model. The null hypothesis assumed the absence of significant odontometric dimorphism between sexes.

## Materials and methods

### Study setting and ethical considerations

This retrospective, cross-sectional investigation was carried out using records from the Outpatient Clinics of the Departments of Paediatric Dentistry and Orthodontics, Faculty of Dentistry, Tanta University, Egypt. Ethical approval was obtained from the Faculty Research Ethics Committee (approval code: R-PED-5-24-3108). Written informed consent to participate was obtained from all participants and/or their legal guardians at the time of record and dental cast collection. All procedures were conducted in accordance with the principles of the Declaration of Helsinki and its later revisions.

### Study sample and eligibility criteria

Dental casts from Egyptian adolescents aged 12 to 19 years were retrieved from the institutional archives. An initial pool of 315 paired maxillary and mandibular models was screened for eligibility. Casts were included if permanent teeth from the right first molar to the left first molar were fully erupted and displayed normal morphology without evidence of occlusal irregularities or developmental anomalies.

Models were excluded when clinical records or cast examination revealed parafunctional habits (e.g., bruxism), mouth breathing, or periodontal pathology. Additional exclusion criteria comprised dental crowding, malalignment, variations in tooth number (congenitally missing, retained primary, or supernumerary teeth), proximal caries, extensive restorations, enamel hypoplasia, or the presence of fixed or provisional crowns that could compromise measurement accuracy. Participants with a history of orthognathic surgery, active orthodontic treatment, cleft palate, palatal asymmetry, anterior or posterior crossbite or significant sagittal malocclusion were also excluded. Casts presenting technical imperfections, including air bubbles, fractures, or dimensional distortion, were omitted.

**Fig. 1:**
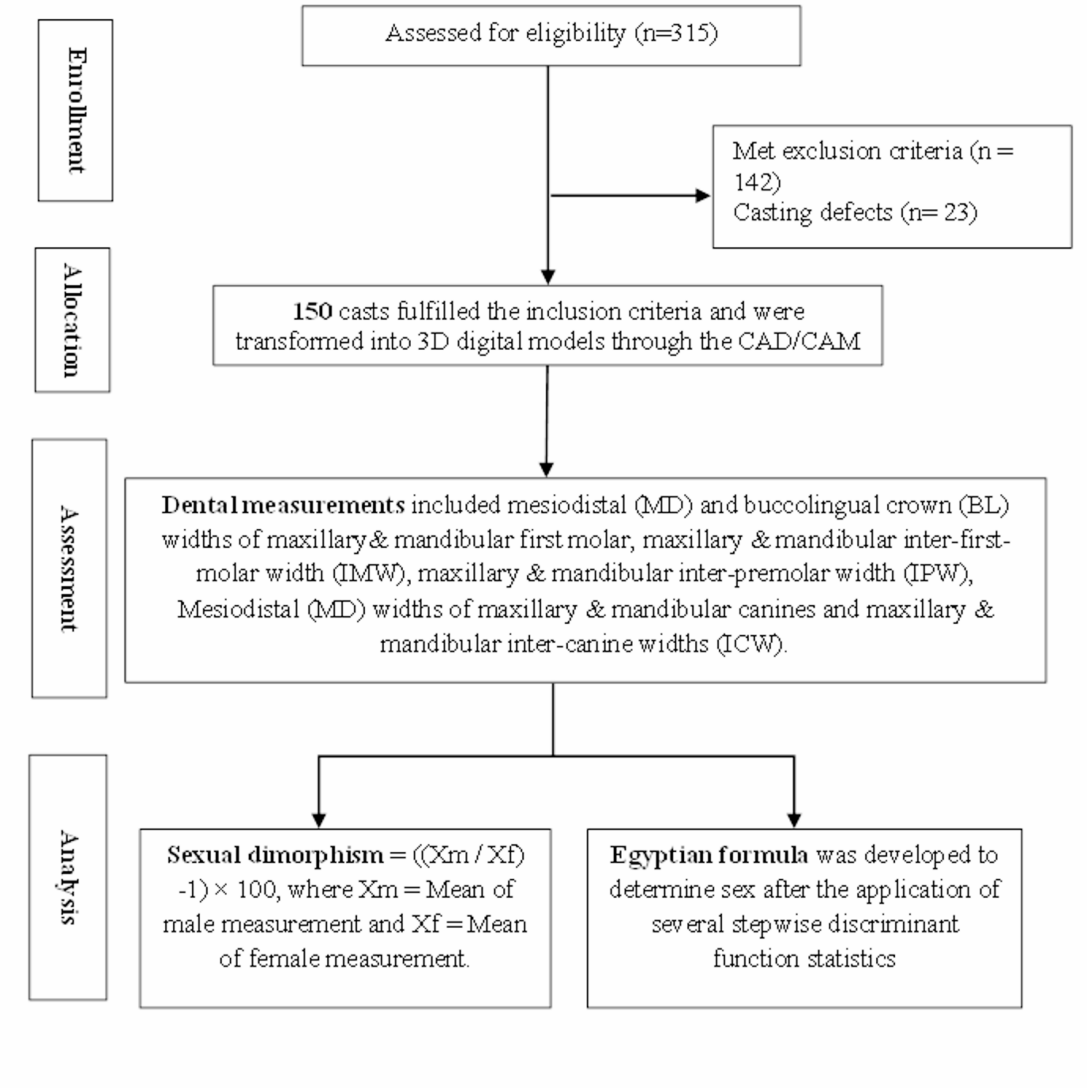
Flow chart explaining the methodological steps utilized in the study

Following the screening process, 165 models were excluded, resulting in a final study sample of 150 eligible dental casts. The selection and screening process is illustrated in the flow diagram (Fig. 1).

### Sample size calculation

Sample size estimation and power analysis were performed using Epi Info™ software (version 7.2.7; Centers for Disease Control and Prevention, Atlanta, GA, USA). The calculation was based on a 95% confidence level, an expected classification accuracy of sex identification of 75%, and a permissible error margin of 7%. The expected classification accuracy of 75% was selected based on previously published odontometric discriminant function studies [18, 19], in which sex classification accuracy typically ranges between approximately 65% and 75% for single-population stepwise multivariate models. This value was therefore considered a conservative upper-bound estimate of achievable performance for odontometric sex estimation using linear discriminant analysis in comparable populations. The minimum required sample was calculated as 147 casts; this number was increased to 150 to account for potential data loss and to enhance the robustness of the statistical analysis.

### Digital model acquisition

**Fig. 2:**
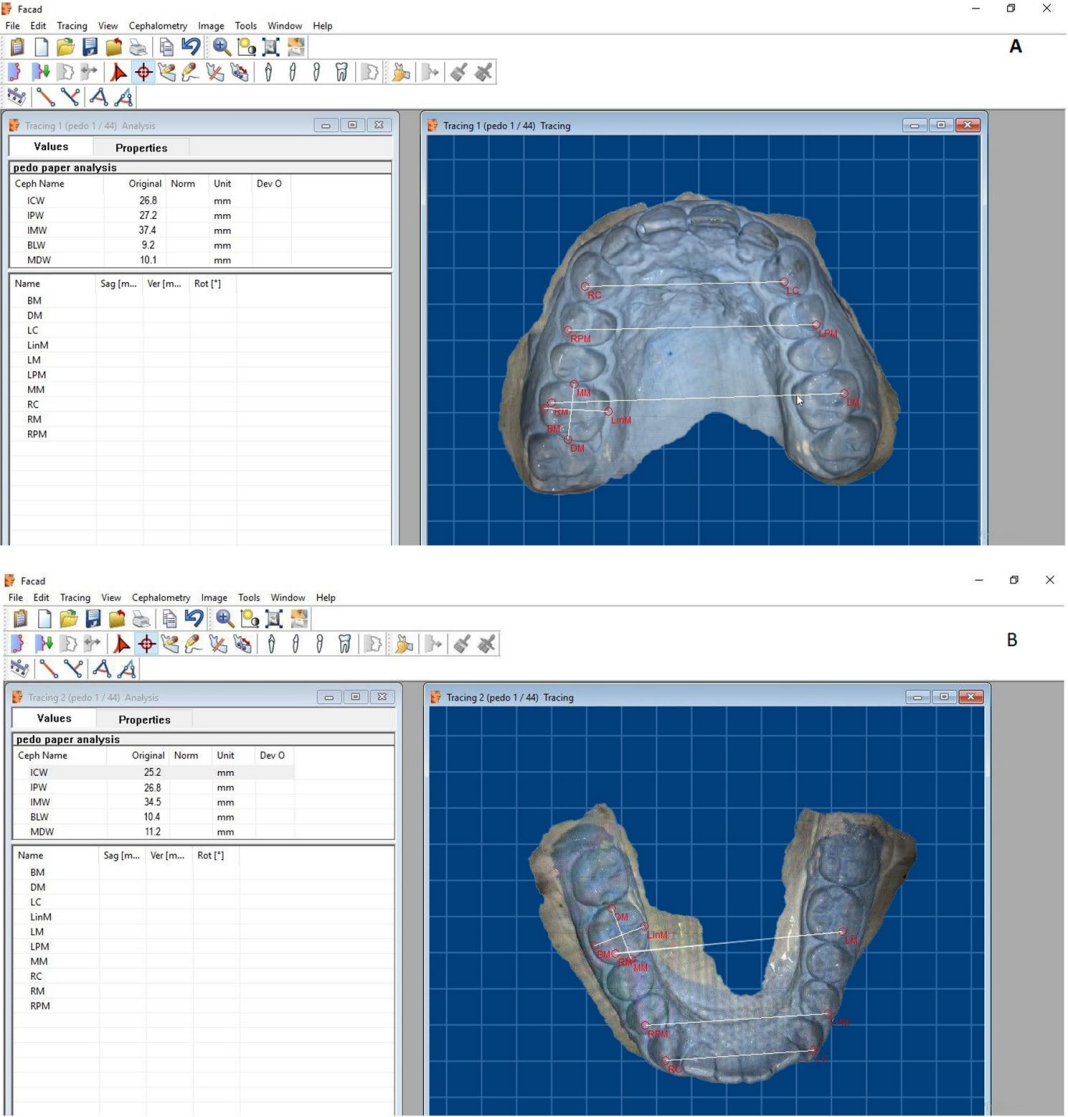
Measurements on the digital cast. **A**: ICW, intercanine width; IPW, interpremolar width; IMW, intermolar width; BLW, buccolingual crown width of the first molar; MDW, mesiodistal crown width of the first molar in the upper arch. **B**: ICW, IPW, IMW, BLW, MDW in the lower arch

Dental casts were digitized using a Helios 500 optical scanner (Changzhou Sifary Medical Technology Co., China) at a 1:1 scale. According to the manufacturer’s specifications, the scanner provides a full-arch accuracy of approximately 20 μm (0.02 mm) and a single-tooth accuracy of ≤ 10 μm. Also, Helios 500 achieves optimal precision at a scanning distance of approximately 5 mm [20]. This level of precision enables high-resolution three-dimensional capture of dental surface morphology suitable for odontometric analysis. The resulting three-dimensional datasets were exported in Standard Tessellation Language (STL) format. These files were subsequently imported into Facad^®^ Ortho tracing software (version 3.10; Ilexis AB, Linköping, Sweden) for two-dimensional visualization and metric assessment (Fig. 2). A customized analysis protocol was developed in the software environment to standardize odontometric assessment. Predetermined anatomical landmarks corresponding to the selected odontometric variables were digitally identified on the scanned casts, after which the software automatically generated the corresponding linear measurements. This standardized workflow helped minimize variability in manual measurements and enhance the reproducibility and consistency of the odontometric analysis.

### Odontometric measurements

All measurements were obtained by a single trained and calibrated examiner (second author), who was blinded to the sex of the subjects to minimize observational bias. Each variable was recorded twice, and the mean of the two readings was used for subsequent analyses.

The following variables were recorded on the digital models (Fig. 2):

- Mesiodistal (MD) crown width of maxillary and mandibular first molars: Defined as the maximum distance between the mesial and distal contact points on the crown. Measurements were obtained bilaterally, and the mean value was used for analysis [21–23].
- Buccolingual (BL) crown width of maxillary and mandibular first molars: Defined as the greatest distance between the buccal and lingual crown surfaces. Bilateral measurements were averaged [22].
- Mesiodistal width of maxillary and mandibular canines: Measured as the maximum mesiodistal distance between proximal contact points on each arch [24].
- Intermolar width (IMW): The linear distance between the mesiobuccal cusp tips of corresponding first molars in opposing quadrants [25].
- Interpremolar width (IPW): The distance between the buccal cusp tips of the corresponding first premolars across the arch [26].
- Intercanine width (ICW): The straight-line distance between the cusp tips of the right and left maxillary canines [25].

All odontometric data were recorded in a coded spreadsheet to ensure subject confidentiality. Demographic variables, including age and sex, were extracted from institutional patient records.

### Statistical analysis

Statistical processing was performed using SPSS software (version 19.0; IBM Corp., Chicago, IL, USA). All continuous variables followed the normal distribution (assessed using the Shapiro-Wilk test, kurtosis coefficients, histograms, and Q-Q plots). Descriptive statistics, including means and standard deviations, were calculated for all variables. To evaluate intra-examiner reliability, 30 digital models were randomly selected and remeasured after a two-week interval. Measurement consistency was assessed using the intraclass correlation coefficient (ICC), based on a two-way mixed-effects model, absolute agreement type. The ICC was interpreted according to the classification proposed by Koo and Li, [27] whereby values below 0.50 indicate poor reliability, values between 0.50 and 0.75 indicate moderate reliability, values between 0.75 and 0.90 indicate good reliability, and values above 0.90 indicate excellent reliability.

Comparisons between males and females were conducted using the independent samples t-test. Mean differences with their 95% confidence intervals (CI) as well as Cohen’s d effect size were calculated to assess the magnitude of sex-related differences in the studied odontometric measurements. Cohen’s d values were interpreted as very small (d < 0.2), small (0.2 ≤ d < 0.5), medium (0.5 ≤ d < 0.8), or large (d ≥ 0.8) according to conventional benchmarks proposed by Cohen, [28].

The degree of sexual dimorphism was expressed as the percentage difference between male and female mean values, calculated using the formula proposed by Garn et al. [27]:

$$\begin{aligned}&\mathbf{Percentage\:of\:sexual\:dimorphism}\\&\mathbf{=}\mathbf{\left(\left(X_{m}/X_{f}\right)-1\right)}\mathbf{\times{100}}\end{aligned}$$

where $\:{X}_{m}$represents the male mean and $\:{X}_{f}$ the female mean.

Discriminant function analysis (DFA) was performed to identify the odontometric variables with the greatest discriminatory power and to develop a predictive model for sex estimation. The assumptions of homogeneity of variance-covariance matrices were assessed using Box’s M test, and multivariate normality was examined to ensure the validity of the model. The model was developed using a forward stepwise selection procedure (entry criterion: F ≥ 3.84; removal criterion: F ≤ 2.71). Wilks’ lambda was used to assess the contribution of each variable to sex discrimination, with smaller values indicating stronger discriminative ability. Eigenvalues, canonical correlation coefficients, and classification function coefficients were calculated to evaluate the performance of the discriminant model. Statistical significance was set at *p* < 0.05. Equal prior probabilities were used in the discriminant function analysis to avoid potential classification bias arising from the slight imbalance between male and female participants in the study sample.

Bootstrap resampling was used to evaluate the internal validity of the discriminant function analysis. The original dataset was resampled with replacement in 1,000 iterations, preserving the original group proportions in each bootstrap sample. This procedure generates two sets within each iteration: the bootstrap training sample (63.2% of the original cases, some appearing more than once) on which the discriminant model was refitted; and the bootstrap test sample (the remaining approximately 36.8% of cases not selected in that iteration), which served as an independent test set for model evaluation. In each iteration, canonical discriminant function analysis was refitted on the training set, and canonical correlations, standardized canonical coefficients, and classification accuracy were recorded. Model performance was evaluated on the corresponding test set. Classification performance metrics were aggregated across all iterations to compute the mean, standard error, and 95% confidence intervals of each metric across bootstrap resamples. Bootstrap-derived 95% confidence intervals were computed using the bias-corrected and accelerated (BCa) method, which accounts for both bias and skewness in the bootstrap distribution, providing more reliable interval estimates than simple percentile-based approaches. In addition, a leave-one-out cross-validation (LOOCV) procedure was applied to generate an independent estimate of classification accuracy. In LOOCV, each case is classified by the functions derived from all cases other than that case.

## Results

A total of 150 study casts belonging to 71 (47.3%) adolescent females and 79 (52.7%) adolescent males with a mean age of 15.84 *±* 0.32 and 15.63 *±* 0.45 years, respectively, with no significant difference, were included in this study. The ICC was 0.87, indicating good intraobserver reliability for the tooth measurements. The sample distribution between males and females was considered in the discriminant function analysis, in which equal prior probabilities were applied to ensure unbiased classification performance.

### Comparison of odontometric variables between sexes

As shown in Table 1, the mean mesiodistal width of the upper first molar was 10.12 *±* 0.62 for males and 8.03 *±* 0.58 for females also, its mean buccolingual width was 10.23 *±* 0.61 for males and 8.06 *±* 0.64 for females with highly statistically significant differences (*p* < 0.001).

**Table 1:** Comparison of the mean value of upper and lower measured parameters according to sex

Variables	Males	Females	Mean difference (95% CI)	Cohen’s D (95% CI)	t	*P*
Mesiodistal upper first molar	10.12 *±* 0.62	8.03 *±* 0.58	2.09 (1.84 to 2.34)	3.49 (2.99 to 4.15)	16.538	< 0.001
Buccolingual upper first molar	10.23 *±* 0.61	8.06 *±* 0.64	2.17 (1.91 to 2.43)	3.49 (2.96 to 4.14)	16.549	< 0.001
Mesiodistal lower first molar	10.77 *±* 0.78	9.29 *±* 0.69	1.48 (1.12 to 1.84)	1.73 (1.27 to 2.23)	8.190	< 0.001
Buccolingual lower first molar	9.66 *±* 0.69	8.44 *±* 0.92	1.22 (0.87 to 1.56)	1.49 (1.11 to 1.95)	7.071	< 0.001
Upper intermolar width	38.95 *±* 1.75	35.05 *±* 1.01	3.90 (3.30 to 4.50)	2.72 (2.08 to 3.70)	12.924	< 0.001
Lower intermolar width	34.44 *±* 2.01	32.43 *±* 2.12	2.00 (1.14 to 2.87)	0.97 (0.58 to 1.53)	4.601	< 0.001
Upper inter premolar width	33.26 *±* 2.67	29.17 *±* 1.73	4.09 (3.14 to 5.04)	1.81 (1.38 to 2.40)	8.605	< 0.001
Lower inter premolar width	32.71 *±* 2.75	29.11 *±* 1.57	3.60 (2.66 to 4.54)	1.61 (1.07 to 2.38)	7.620	< 0.001
Upper inter-canine width	26.31 *±* 1.56	24.06 *±* 1.59	2.24 (1.58 to 2.90)	1.42 (1.00 to 1.93)	6.751	< 0.001
Lower inter-canine width	26.11 *±* 1.59	23.96 *±* 1.37	2.15 (1.53 to 2.77)	1.45 (1.02 to 1.93)	6.861	< 0.001
Mesiodistal upper canine	7.76 *±* 0.66	6.67 *±* 0.66	1.09 (0.81 to 1.37)	1.66 (1.24 to 2.22)	7.852	< 0.001
Mesiodistal lower canine	6.70 *±* 0.78	5.78 *±* 0.67	0.92 (0.62 to 1.22)	1.26 (0.85 to 1.81)	5.999	< 0.001

*CI* Confidence interval

The upper intermolar widths for males and females were 38.95 *±* 1.75 and 35.05 *±* 1.01 respectively while the upper inter-premolar widths were 33.26 *±* 2.67 and 29.17 *±* 1.73 for males and females respectively with highly statistically significant differences (*p* < 0.001).

In addition, the mean mesiodistal widths of upper and lower canines, lower inter-premolar width, and lower intermolar width were found to be higher in males than females with statistically significant differences (*p* < 0.001) (Table 1).

### Degree of sexual dimorphism

**Fig. 3:**
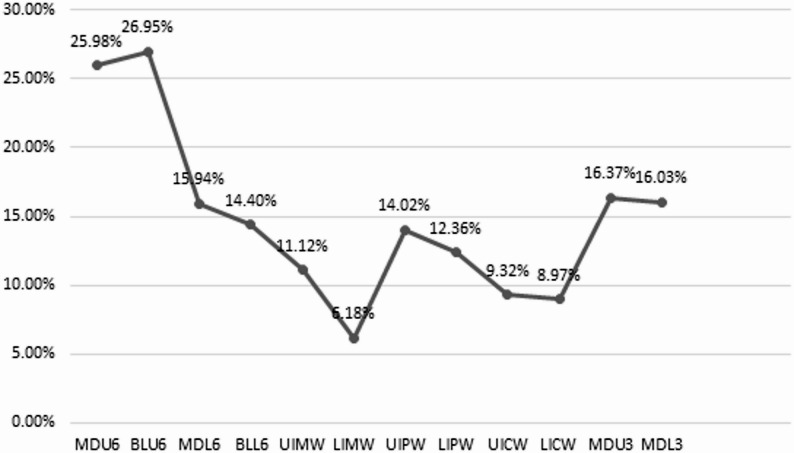
Percentages of sexual dimorphism of measured parameters

The highest sexual dimorphism percentage was observed for the buccolingual width of the upper first molar (26.95%), followed by its mesiodistal width (25.98%). In contrast, the lowest percentage was recorded for the lower intermolar width (6.18%) (Table 2; Fig. 3).

**Table 2:** Percentage of Sexual Dimorphism of the measured parameters

Variables	Dimorphism Percentage
Mesiodistal upper first molar (MDU6)	25.98%
Buccolingual upper first molar (BLU6)	26.95%
Mesiodistal lower first molar (MDL6)	15.94%
Buccolingual lower first molar (BLL6)	14.4%
Upper intermolar width (UIMW)	11.12%
Lower intermolar width (LIMW)	6.18%
Upper inter premolar width (UIPW)	14.02%
Lower inter premolar width (LIPW)	12.36%
Upper inter-canine width (UICW)	9.32%
Lower inter-canine width (LICW)	8.97%
Mesiodistal upper canine (MDU3)	16.37%
Mesiodistal lower canine (MDL3)	16.03%

### Development of the discriminant formula

Prior to stepwise discriminant function analysis, all odontometric variables were examined using univariate tests of equality of group means to evaluate their suitability for model entry. As shown in Table 3 (Step 0), all variables met the entry criterion (*p* < 0.001). Subsequently, a stepwise discriminant function analysis was performed to identify the optimal subset of variables for sex classification. Equal prior probabilities were assumed for both groups, and classification was based on pooled within-group covariance matrices. Variable selection was performed using Wilks’ lambda, with entry and removal criteria set at *p* < 0.05 and *p* > 0.10, respectively.

**Table 3:** Univariate Wilks’ lambda tests for odontometric variables prior to stepwise discriminant analysis

Variables	Wilk’s Lambda	F	*p*
Mesiodistal upper first molar	0.243	237.498	< 0.001
Buccolingual upper first molar	0.243	273.868	< 0.001
Mesiodistal lower first molar	0.567	67.082	< 0.001
Buccolingual lower first molar	0.638	49.994	< 0.001
Upper intermolar width	0.345	167.022	< 0.001
Lower intermolar width	0.806	21.172	< 0.001
Upper inter-premolar width	0.543	74.052	< 0.001
Lower inter-premolar width	0.602	58.071	< 0.001
Upper inter-canine width	0.659	45.573	< 0.001
Lower inter-canine width	0.651	47.077	< 0.001
Mesiodistal upper canine	0.588	61.653	< 0.001
Mesiodistal lower canine	0.710	35.992	< 0.001

The steps of the stepwise model are depicted in the supplementary table S1. The stepwise procedure retained four predictor variables in the final discriminant model: mesiodistal width of the maxillary first molar, buccolingual width of the maxillary first molar, maxillary intermolar width, and maxillary interpremolar width. These variables demonstrated the strongest discriminatory capability for sex estimation in the studied population. Interpretation of the discriminant function was supported by the structure matrix (Supplementary Table S2), which showed that the four variables had the highest correlations with the discriminant function.

Group centroids representing the mean discriminant score for each sex were generated from the final model. The male group centroid was 2.950, whereas the female group centroid was − 2.950, with a cutoff value of zero used for classification (Table 4). Scores below zero were classified as female, whereas scores above zero were classified as male.

**Table 4:** Standardized and unstandardized canonical discriminant function coefficients of the final model

Variables	Unstandardized coefficients	Standardized coefficients	Classification function coefficients	
			Males	Females
Buccolingual upper first molar	0.906	0.564	24.235	19.071
Mesiodistal upper first molar	0.855	0.511	22.604	17.732
Upper intermolar width	0.293	0.419	18.158	16.487
Upper inter-premolar width	0.198	0.446	8.351	7.223
Constant	-33.055	-	-731.447	-542.993

Wilks’ Lambda = 0.107, Chi square = 191.880, *p* < 0.001. Eigenvalue = 8.898, Canonical correlation = 0.948

Classification function coefficients result for males = 97.8%

Classification function coefficients result for females = 100%

Discriminant score = 0.855 (Mesiodistal upper first molar) + 0.906 (Buccolingual upper first molar) + 0.293 (Upper intermolar width) + 0.198 (Upper inter premolar width) -33.055

Group centroids: 2.950 for males and − 2.950 for females

Cutoff point = 0; If the discriminant score > cutoff point, the individual is male

Table 4 presents the canonical discriminant function coefficients and unstandardized coefficients for the retained predictor variables. The discriminant score was calculated by multiplying each odontometric variable by its corresponding unstandardized coefficient and adding the constant value, according to the following equation:

$$\begin{aligned}&\mathbf{Discriminant\:score}\\&=\mathbf{0.855\left(Mesiodistal\:upper\:first\:molar\right)}\\&\mathbf{+0.906\left(Buccolingual \:upper\:first\:molar\right)}\\&\mathbf{+0.293\left(Upper\:intermolar\:width\right)}\\&\mathbf{+0.198\left(Upper\:inter\:premolar\:width\right)}\mathbf{-33.055}\end{aligned}$$

The developed Egyptian discriminant model demonstrated a statistically significant contribution to sex estimation (χ² = 191.880, *p* < 0.001). Application of the formula to the study sample yielded classification accuracies of 97.8% for males and 100% for females, indicating promising discriminatory performance within the studied population (Table 5).

**Table 5:** Model performance

Metrics	Estimate (95% confidence interval)
Sensitivity (%)	97.78 (88.23 to 99.9)
Specificity (%)	100.00 (92.13 to 100.00)
Accuracy (%)	98.89 (93.96 to 99.97)
Kappa coefficient	0.978 (0.934 to 1.000)

Internal validation by bootstrapping (1000 resamples) was conducted. Bootstrapped results demonstrated robust and stable classification performance across resampled datasets. Canonical coefficients showed minimal variability across resamples. The classification accuracy obtained through bootstrap resampling was comparable to the original model, with only minor attenuation, suggesting limited overfitting. The LOOCV procedure yielded comparable performance metrics (Supplementary Tables S3, S4, and S5).

## Discussion

Accurate determination of sex represents a fundamental component of the biological profile in forensic identification. Dental tissues, due to their structural durability and resistance to postmortem alteration, provide a reliable source of morphological and metric information for this purpose [29, 30].

The present study is distinguished by its combined evaluation of individual tooth dimensions and transverse dental arch measurements across both jaws. This integrated approach enhances practical applicability, particularly in scenarios where individual teeth may be absent but arch dimensions remain measurable. Moreover, transverse arch growth is largely completed before late adolescence, and intercanine width has been reported to stabilize after early teenage years, supporting the suitability of this age group for odontometric assessment [31].

Participants were selected within the 12–19-year age range to minimize the influence of attrition, restorative intervention, and age-related morphological changes. At this stage, crown formation is complete and dimensional variability is relatively limited, allowing more reliable measurement of inherent tooth size differences [21, 32]. The aforementioned age range was found to be comparable to that of Al-Rifaiy et al. [33] (15–18 years) and Rao et al. [34] (15–21 years).

Measurements in this study were obtained from digitized dental models, which offer several methodological advantages over direct intraoral or stone cast assessments. Digital models allow precise, repeatable measurements, simplified data storage, and reduced risk of physical degradation or loss of records, contributing to improved analytical reliability [6, 35].

The results of this study rejected the null hypothesis as there is a sex-based teeth dimorphism in Egyptian adolescents. The findings of the present study generally indicated that male Egyptian participants had considerably greater dental and arch measures than females; these larger dental dimensions in males compared to females may be ascribed to increased enamel and dentin thickness in males [36–38]. Narang et al. [39] elucidated that the X chromosome promotes amelogenesis, whereas the Y chromosome enhances the mitotic potential of tooth germ and stimulates dentinogenesis, leading to greater enamel and dentin thickness in men compared to women.

In the current study, the predictor variables used in the discriminant Egyptian formula were the mesiodistal upper first molar, buccolingual upper first molar, upper intermolar width, and upper inter-premolar width. The upper first molar’s buccolingual width (26.95%) and mesiodistal width (25.98%) showed the highest percentages of sexual dimorphism, which was greater than the mesiodistal width of upper and lower canines (16.37% and 16.03%, respectively). This was in line with Reddy et al. [40] who evaluate the mesiodistal and buccolingual dimensions of first permanent molars and canines for the determination of sexual dimorphism in Indian subjects. In contrast to earlier studies, which focused primarily on the premolars and canines, largely ignoring the first molar’s importance as a sex predictor [4].

The maxillary first molars are the largest and most robust teeth due to their substantial crown size and superior stability provided by their multiple roots. The maxillary first molars commence calcification at birth and erupt approximately at 6 years of age. Due to its completion before skeletal maturity, it serves as a more dependable indicator for sex estimation [41]. Previous research indicated a statistically significant sexual dimorphism in the maxillary first molar [39, 42, 43].

The present study findings revealed that Egyptian males have considerably greater inter-first molar widths compared to females; this may be attributed to the greater size of the male skeleton in comparison to the female skeleton [29]. This agreed with Al-Zubair, [44] study on the Yemeni population, also, Rao and Kiran, [1] achieved analogous results among the Telangana people. Moreover, these results also aligned with those from studies conducted on Chinese [45] and Saudi [46]cohorts. In addition, Bano and Babu, [47] study in the Indian population confirmed that the maxillary inter-first molar width serves as a more precise indicator of sex than the maxillary inter-canine width.

Concerning the maxillary inter-first premolar width in the present study, it was considerably greater in males compared to females; this coincided with the findings of Grewal et al. [26] who reported that the inter-first premolar width serves as an effective predictor of sex with substantial accuracy. Also, these results agreed with Sharif et al. [29] who investigated sex estimation from maxillary arch measurements in 1410 adults Egyptian population aged 18–55 years. However, their study was limited to maxillary parameters and an adult population. In contrast, the present research evaluated a broader set of odontometric variables from both maxillary and mandibular casts, focusing specifically on adolescents, a group that had not been adequately addressed in previous Egyptian studies. This broader and more targeted approach allowed us to establish a new odontometric formula specifically for Egyptian adolescents.

The discriminant Egyptian formula contributed significantly to estimating sex (X^2^ = 191.880, *p* < 0.001), and the result of classification function coefficients was 97.8% and 100% for males and females, respectively; this level of accuracy was greater than that obtained by previous studies using different indices [4, 48, 49]. Hence, this indicates that the discriminant formula obtained can effectively distinguish sexual dimorphism in the Egyptian population. However, while internal validation was performed using bootstrapping and also LOOCV, the risk of overfitting cannot be ruled out, as these methods remain subject to optimism bias when applied to the same dataset used for model development. Furthermore, the relatively modest sample size may increase susceptibility to overfitting, as discriminant models derived from smaller samples tend to inflate classification accuracy beyond what would be observed in larger, independent datasets. Thus, the very high classification accuracy reported in this study may partially reflect model overfitting. Consequently, external validation using independent datasets - that were not used in model development - is necessary to confirm the robustness and validity of the proposed model.

It is well established that odontometric parameters vary considerably among different populations due to genetic, environmental, and ethnic factors. Therefore, one limitation of the present study is that the proposed discriminant function equation may not be directly applicable to other populations without prior external validation. Additionally, the study sample was recruited from patients attending the Departments of Paediatric Dentistry and Orthodontics, which may introduce a degree of selection bias. Although strict inclusion criteria were applied to ensure normal occlusion and typical dental morphology, the findings may not be fully representative of the broader Egyptian adolescent population. Future studies utilizing larger and more randomly selected population-based samples from multiple different regions are therefore recommended to validate and enhance the generalizability of the proposed discriminant model.

Furthermore, although participants were selected within a relatively narrow adolescent age range, minor variations in dental maturation may still occur during this developmental period and could potentially influence odontometric measurements. Age-related developmental variability should therefore be considered when interpreting the results. Future research incorporating larger samples and age-stratified analyses would help clarify the potential influence of dental maturation on the accuracy of sex estimation.

Another potential limitation relates to technical factors associated with digital scanning and image processing. Errors occurring during the acquisition or processing of three-dimensional digital models may introduce minor distortions that could affect the accuracy of odontometric measurements. Additionally, inter-examiner reliability was not assessed in the present study, as all measurements were performed by a single examiner. Although this approach helped maintain measurement consistency, the evaluation of both intra- and inter-examiner reliability in future studies would further enhance the methodological rigor and reproducibility of odontometric analyses.

## Conclusion

Egyptian adolescent males exhibited significantly greater mesiodistal and buccolingual dimensions of the maxillary first molars, as well as larger transverse maxillary widths, compared with females. Measurements of intermolar and interpremolar widths, together with first molar dimensions, demonstrated promising discriminatory capability for sex estimation. The predictive model developed in this study may provide a useful population-specific adjunctive tool for forensic and anthropological applications. Nevertheless, further external validation using independent samples and populations from different ethnic and geographic backgrounds is recommended to confirm its broader applicability and generalizability.

## Supplementary Information

Supplementary Material 1.

## Data Availability

On reasonable request, the datasets utilized and/or analyzed during the present study are accessible from the corresponding author.
