# Supplementary material for: Digital odontometric analysis for sex estimation in Egyptian adolescents
Source: BMC Oral Health. 2026 Aug 8;26:1384. doi: 10.1186/s12903-026-09486-x (PMC13452096; doi:10.1186/s12903-026-09486-x)
Supplement: Supplementary file 1 [file 12903_2026_9486_MOESM1_ESM.docx]

**Supplementary material**

**Table S1. Stepwise discriminant analysis: variables entered at each step in the model**

| Step | | Tolerance | Sig. of F to Remove | Wilks' Lambda |
| --- | --- | --- | --- | --- |
| 1 | Buccolingual upper first molar | 1.000 | <0.001 |  |
| 2 | Buccolingual upper first molar | 0.973 | <0.001 | 0.243 |
|  | Mesiodistal upper first molar | 0.973 | <0.001 | 0.243 |
| 3 | Buccolingual upper first molar | 0.953 | <0.001 | 0.196 |
|  | Mesiodistal upper first molar | 0.973 | <0.001 | 0.180 |
|  | Upper inter premolar width | 0.977 | <0.001 | 0.158 |
| 4 | Buccolingual upper first molar | 0.949 | <0.001 | 0.147 |
|  | Mesiodistal upper first molar | 0.970 | <0.001 | 0.139 |
|  | Upper inter premolar width | 0.976 | <0.001 | 0.130 |
|  | Upper intermolar width | 0.990 | <0.001 | 0.127 |

**Table S2. Structure matrix of canonical discriminant function**

| Variables | Function1 |
| --- | --- |
| Buccolingual upper first molar | 0.612 |
| Mesiodistal upper first molar | 0.612 |
| Upper intermolar width | 0.478 |
| Upper inter premolar width | 0.318 |
| Lower inter premolar width^a^ | 0.246 |
| Mesiodistal lower canine^a^ | 0.132 |
| Lower inter-canine width^a^ | 0.132 |
| Buccolingual lower first molar^a^ | 0.126 |
| Mesiodistal lower first molar^a^ | 0.122 |
| Mesiodistal upper canine^a^ | 0.118 |
| Lower intermolar width^a^ | 0.098 |
| Upper inter-canine width^a^ | 0.081 |
| Pooled within-groups correlations between discriminating variables and standardized canonical discriminant functions  Variables are ordered by absolute correlation with the discriminant function. Variables marked with superscript a were not used in the analysis as they were excluded from the final stepwise model, but are shown for interpretative completeness. | |

**Table S3. Summary of bootstrapped results for the canonical discriminant function coefficients of the final selected model**

| **Canonical Discriminant Function Coefficients** | | | | | |
| --- | --- | --- | --- | --- | --- |
| **Variables** | **Unstandardized original Coefficient** | **Bootstrap results** | | | |
|  |  | **Bias** | **SE** | **BCa 95% Confidence Interval** | |
|  |  |  |  | **Lower** | **Upper** |
| Buccolingual upper first molar | 0.906 | 0.028 | 0.152 | 0.565 | 1.343 |
| Upper intermolar width | 0.293 | 0.019 | 0.102 | 0.108 | 0.574 |
| Upper inter premolar width | 0.198 | 0.008 | 0.062 | 0.070 | 0.349 |
| Mesiodistal upper first molar | 0.855 | 0.026 | 0.155 | 0.532 | 1.268 |
| Constant | -33.055 | -1.461 | 4.753 | -41.822 | -28.080 |
| BCa: Bias Corrected Bootstrap Confidence Intervals; SE: Standard error of bias | | | | | |
| Bootstrap results are based on 1000 bootstrap samples | | | | | |

**Table S4. Summary of bootstrapped results for the classification function coefficients of the final selected model.**

| **Classification Function Coefficients** | | | | | | |
| --- | --- | --- | --- | --- | --- | --- |
| **Variables** | **sex** | **Coefficient** | **Bootstrap** | | | |
|  |  |  | **Bias** | **Std. Error** | **BCa 95% Confidence Interval** | |
|  |  |  |  |  | **Lower** | **Upper** |
| Buccolingual upper first molar | Male | 24.235 | 2.326 | 8.566 | 9.842 | 52.681 |
|  | Female | 19.071 | 1.880 | 7.374 | 6.880 | 42.608 |
| Upper intermolar width | Male | 18.158 | 2.275 | 5.636 | 10.357 | 43.104 |
|  | Female | 16.487 | 2.039 | 4.899 | 9.780 | 38.897 |
| Upper inter premolar width | Male | 8.351 | 1.034 | 3.208 | 3.352 | 19.662 |
|  | Female | 7.223 | .906 | 2.758 | 2.717 | 16.906 |
| Mesiodistal upper first molar | Male | 22.604 | 1.456 | 6.760 | 11.473 | 41.544 |
|  | Female | 17.732 | 1.084 | 5.846 | 7.941 | 33.302 |
| Constant | Male | -731.447 | -81.210 | 192.997 | -1106.169 | -580.444 |
|  | Female | -542.993 | -60.931 | 142.357 | -807.745 | -433.831 |
| BCa: Bias Corrected Bootstrap Confidence Intervals; SE: Standard error of bias | | | | | | |
| Bootstrap results are based on 1000 bootstrap samples | | | | | | |

**Table S5. Averaged performance of bootstrapped results**

| **Metrics** | **Bootstrap validation*^a^*** | **Leave‑one‑out cross‑validation** |
| --- | --- | --- |
|  | **Estimate (95% confidence interval)** | **Estimate** |
| Sensitivity (%) | 97.79 (93.62 to 100.00) | 97.78 |
| Specificity (%) | 100.00 (100.00 to 100.00) | 100.00 |
| Accuracy (%) | 98.87 (96.67 to 100.00) | 98.89 |
| Kappa coefficient | 0.978 (0.933 to 1.000) | 0.978 |
| ***^a^*** Based on 1000 bootstrap resamples | | |
